# The DSM-5 introduction of the Social (Pragmatic) Communication Disorder as a new mental disorder: a philosophical review

**DOI:** 10.1007/s40656-021-00460-0

**Published:** 2021-09-24

**Authors:** M. Cristina Amoretti, Elisabetta Lalumera, Davide Serpico

**Affiliations:** 1 Department of Classics, Philosophy and History (DAFIST), Philosophy Section, University of Genoa, Via Balbi 4, 16126 Genoa, Italy; 2 Department for Life Quality Studies (QUVI), University of Bologna, Corso di Augusto 237, 47921 Rimini, Italy

**Keywords:** Social (pragmatic) communication disorder (SPCD), Autism spectrum disorder (ASD), Mental disorders, Psychiatric nosology, DSM-5

## Abstract

The latest edition of the Diagnostic and Statistical Manual of Mental Disorders (DSM-5) included the Social (Pragmatic) Communication Disorder (SPCD) as a new mental disorder characterized by deficits in pragmatic abilities. Although the introduction of SPCD in the psychiatry nosography depended on a variety of reasons—including bridging a nosological gap in the macro-category of Communication Disorders—in the last few years researchers have identified major issues in such revision. For instance, the symptomatology of SPCD is notably close to that of (some forms of) Autism Spectrum Disorder (ASD). This opens up the possibility that individuals with very similar symptoms can be diagnosed differently (with either ASD or SPCD) and receive different clinical treatments and social support. The aim of this paper is to review recent debates on SPCD, particularly as regards its independence from ASD. In the first part, we outline the major aspects of the DSM-5 nosological revision involving ASD and SPCD. In the second part, we focus on the validity and reliability of SPCD. First, we analyze literature on three potential validators of SPCD, i.e., etiology, response to treatment, and measurability. Then, we turn to reliability issues connected with the introduction of the *grandfather clause* and the use of the concepts of *spectrum* and *threshold* in the definition of ASD. In the conclusion, we evaluate whether SPCD could play any role in contemporary psychiatry other than that of an independent mental disorder and discuss the role that non-epistemic factors could play in the delineation of the future psychiatry nosography.

## Introduction

The latest edition of the Diagnostic and Statistical Manual of Mental Disorders, DSM-5 (American Psychiatric Association, 2013) has introduced the Social (Pragmatic) Communication Disorder (SPCD) as a new category of the psychiatry nosography. This disorder, which has been included in the macro-category of Communication Disorders (CDs), is characterized by a primary difficulty with pragmatic abilities broadly conceived. Typical symptoms of SPCD are deficits in using communication for social purposes, impairment of the ability to change communication to match the context, and difficulties in following the conversational rules (American Psychiatric Association, 2013, p. 47).

Within the transition from DSM-IV to DSM-5, there were various reasons for introducing a new disorder like SPCD into the Manual. Among them, one reason was to pick out individuals affected with language and communication difficulties that do not fall within the range of the typical Specific Language Impairments (SLI)—indeed, individuals with SPCD may have normal phonological processing, vocabulary, and higher-order grammatical and semantic skills (Adams & Bishop, 1989; Bishop & Rosenbloom, 1987; Ketelaars & Embrechts, 2017; Leyfer et al., 2008; Rapin & Allen, 1983).

Other reasons behind the introduction of SPCD probably connect to major changes that were made, for partially independent reasons, to the category of Autistic Disorder and to the macro-category of Pervasive Developmental Disorders (PDDs). The transition from DSM-IV to DSM-5 involved some sort of simplification of the diagnostic criteria for autism-related disorders (Solomon, 2017b) and led to the elimination of a variety of developmental disorders, including Asperger’s Disorder, Childhood Disintegrative Disorder, and Rett’s Disorder. The new category of Autism Spectrum Disorder (ASD) thus replaced the DSM-IV macro-category of PDDs and its disorders altogether.^1^ Within this revision, SPCD ended up grouping together subjects who would have had a DSM-IV autism-related diagnosis but who do not meet a DSM-5 diagnosis of ASD (Brukner-Wertman et al., 2016; Flax et al., 2019; Regier et al., 2013; Swineford et al., 2014).^2^

Although this nosological revision might sound plausible and somewhat necessary, research on SPCD has identified major issues connected with the introduction of SPCD as an independent category. For instance, since the boundaries between ASD, SPCD, and other communication disorders are not as clear, the revision opened up the possibility that individuals with very similar symptoms can be diagnosed differently (with either ASD or SPCD, or even with no diagnosis at all; Swineford et al., 2014). It is also worth mentioning that the DSM-5 revision has significantly narrowed down the autism category (in contrast with the aim of DSM-5 of being more inclusive and covering a broader spectrum of conditions). As Reichow and Volkmar (2018) noted, the revision “decreased the number of clinical criteria considered for a diagnosis [of autism], and limited the ways those criteria are met. […] In the DSM-IV, more than 2000 combinations of features could lead to a diagnosis of autism, but with the DSM-5, only 12 can”.^3^ Relatedly, epidemiological studies have pointed at a decrease in the number of diagnoses of ASD and DSM-IV Autistic Disorder, but only a small part (less than one third) of the people that were previously diagnosed with DSM-IV Autistic Disorder, and now do not meet the criteria of DSM-5 ASD, would qualify for SPCD (Kulage et al., 2019). In brief, the DSM-5 revision has affected the diagnosis of autism and related disorders in a variety of ways, but the full range of epistemological, social, and ethical implications is yet to be fully considered.

In this paper, we review the main existing literature on SPCD to assess the available evidence regarding the independence of SPCD from ASD. Our analysis follows the path of other scholars interested in the epistemology of the DSM, who recently wrote about specific diagnostic categories, including Rachel Cooper on Hoarding Disorder (Cooper, 2015), Miriam Solomon on the Asperger’s Disorder (Solomon, 2017b), and Anke Bueter and Saana Jukola on Hypoactive Sexual Desire Disorder (Bueter & Jukola, 2020).

In the first part, we outline the major aspects of the DSM-5 nosological revision of the two macro-categories of PDDs and CDs, particularly regarding ASD and SPCD, respectively. First, in Sect. 2, we illustrate the transition from the heterogeneous DSM-IV category of PDDs to the comprehensive and unified category of ASD. Second, in Sect. 3, we focus on the introduction of SPCD within the category of CDs; here, we also assess the major differences between ASD and SPCD and identify potential sources of inconsistency or unreliability in the diagnostic process.

In the second part, we review current discussions on the validity and reliability of SPCD. In Sect. 4, we focus on the available data on three major scientific validators: etiology, response to treatment, and measurability. Our main aim in this section is to assess whether research on these aspects support the DSM-5 nosological changes outlined above or suggests any possible alternative interpretation of SPCD. Then, in Sect. 5, we focus on reliability issues, particularly on three aspects of the DSM-5 revision of ASD and SPCD: first, the introduction of the so-called “grandfather clause”; second, the introduction of the concept of *spectrum* in place of the concept of *category*; and third, the introduction of thresholds in the definition of autism disorders. We will argue that these aspects paved the way for nosological ‘grey areas’, inconsistencies in diagnosis, and conceptual shortcomings.

In the conclusion, we discuss whether SPCD could play any role in contemporary psychiatry other than that of an independent mental disorder. We will consider three main options: the reduction of SPCD to a subtype of another disorder; the conceptualization of SPCD as a cluster of symptoms; and its conversion into a research entity.

Towards the end, we discuss the role that non-epistemic factors might (or should) play in the delineation of the future psychiatry nosography, particularly whether it should include SPCD or not. Like many philosophers, historians, and theorists of science have pointed out, it is often the case that nosological decisions in biomedical sciences are made for pragmatic reasons, such as maximizing the patients’ welfare in terms of treatment options and social support. In the case of SPCD, such non-epistemic factors may turn out to be of central importance due to the absence of conclusive epistemic evidence for its inclusion in the psychiatry nosology.

It is worth noting that each edition of the DSM is the product of the interaction of different groups (psychiatrists, psychologists, patients’ advocates, research partners, etc.) pursuing a variety of goals and, for this reason, it cannot be expected to be without limitations or involve no compromises. Nonetheless, a comprehensive epistemological discussion on this topic seems to us essential for preventing shortcomings in future revisions of the two categories of ASD and SPCD. Our aim, however, is not to assess whether SPCD simply ‘exists’ or is a ‘natural category’. Rather, we would like to highlight that the introduction of SPCD in the DSM, together with the revision of PDDs, has generated issues regarding the projectability and generalization capability of the Manual’s categories that are crucial in the diagnostic and treatment processes. In particular, the vague boundaries between the two categories of SPCD and ASD might not allow us to predict reliably the characteristics of individuals belonging to such categories and project findings from one individual to another within the two categories.

## Nosological revisions from DSM-IV and DSM-5: autism-related disorders

In DSM-IV (American Psychiatric Association, 1994) and DSM-IV-TR (American Psychiatric Association, 2000), the category of Autistic Disorder was included among Pervasive Developmental Disorders (PDDs), a macro-category that also comprised Asperger’s Disorder, Childhood Disintegrative Disorder, Rett’s Disorder, and Pervasive Developmental Disorder Not Otherwise Specified (PDD-NOS).

Since the early days, Autistic Disorder was characterized by three classes of symptoms (Kanner, 1943), involving a variety of deficits relating to social interactions and behavior. In DSM-IV, in particular, a diagnosis occurred only if a total of six (or more) items from (1), (2), and (3) were present, with at least two from (1) and one each from (2) and (3) (American Psychiatric Association, 2000, p. 75):

1. Qualitative impairment in social interaction, which includes for instance non-verbal behaviors, the ability to develop relationships with the others, and emotional reciprocity;
2. Qualitative impairments in communication, which includes delay in the development of spoken language and stereotyped or repetitive use of language;
3. Restricted repetitive and stereotyped patterns of behavior, interests, and activities, such as motor mannerisms and inflexible adherence to specific, nonfunctional routines or rituals.

The fifth and latest edition of the DSM, published in 2013 (American Psychiatric Association, 2013), introduced various modifications to the definition, assessment, and categorization of PDDs. In particular, it was introduced a new disorder, namely, Autism Spectrum Disorder (ASD), which replaced the DSM-IV macro-category of PDDs and its disorders altogether.

ASD is characterized as involving two major symptomatic clusters.^4^ Cluster (A) involves *deficits in social communication and social interaction* (DSC, hereafter), such as deficits in social-emotional reciprocity, in nonverbal communicative behaviors, and in the development of relationships. In the textbook’s own words (American Psychiatric Association, 2013, p. 50), the first diagnostic criterion of ASD is:

A. Persistent deficits in social communication and social interaction across multiple contexts, as manifested by the following, currently or by history […]:

1. Deficits in social-emotional reciprocity, ranging, for example, from abnormal social approach and failure of normal back-and-forth conversation; to reduced sharing of interests, emotions, or affect; to failure to initiate or respond to social interactions.
2. Deficits in nonverbal communicative behaviors used for social interaction, ranging, for example, from poorly integrated verbal and nonverbal communication; to abnormalities in eye contact and body language or deficits in understanding and use of gestures: to a total lack of facial expressions and nonverbal communication.
3. Deficits in developing, maintaining, and understanding relationships, ranging, for example, from difficulties adjusting behavior to suit various social contexts; to difficulties in sharing imaginative play or in making friends; to absence of interest in peers. […]

It can be noted that DSC clusters together a variety of symptoms that, in DSM-IV, were included in the first two classes of symptoms characterizing ASD: (1) “qualitative impairment in social interaction” and (2) “qualitative impairment in communication” (see above).^5^

Cluster (B) of ASD, which involves *restricted and repetitive behavior* (RRB, hereafter), remains an independent class of symptoms as it was in DSM-IV (American Psychiatric Association, 2013, p. 50). So, the second diagnostic criterion for ASD is:

B. Restricted, repetitive patterns of behavior, interests, or activities, as manifested by at least two of the following, currently or by history […]:

1. Stereotyped or repetitive motor movements, use of objects, or speech (e.g., simple motor stereotypies, lining up toys or flipping objects, echolalia, idiosyncratic phrases).
2. Insistence on sameness, inflexible adherence to routines, or ritualized patterns of verbal or nonverbal behavior (e.g., extreme distress at small changes, difficulties with transitions, rigid thinking patterns, greeting rituals, need to take same route or eat same food every day).
3. Highly restricted, fixated interests that are abnormal in intensity or focus (e.g., strong attachment to or preoccupation with unusual objects, excessively circumscribed or perseverative interests).
4. Hyper- or hypo-reactivity to sensory input or unusual interest in sensory aspects of the environment (e.g., apparent indifference to pain/temperature, adverse response to specific sounds or textures, excessive smelling or touching of objects, visual fascination with lights or movement).

It can be noted that Cluster B not only includes symptoms that are relatively the same as those included in the third class of symptoms characterizing Autistic Disorder in DSM-IV, that is, (3) “restricted repetitive and stereotyped patterns of behavior, interests, and activities”, but it was also significantly widened with the addition of stereotyped language (criterion 1) and sensory issues (criterion 4).

At the level of symptoms, as Miriam Solomon has commented, the DSM-5 revision of autistic disorders involved some sort of simplification of the diagnostic criteria, in that only two orthogonal dimensions of impairment instead of three are considered in the new edition, namely, DSC, which includes deficits in both social interactions and communication, and RRB (Solomon, 2017b, p. 178).

However, DSM-5 introduced a new parameter aimed at capturing the complexity of the autistic symptomatology, its inter-individual variability, and its fluctuation over time, namely, the assessment of symptoms’ severity,^6^ which is intended to include previously separate diagnostic categories into a dimensional spectrum (American Psychiatric Association, 2013, p. 9).^7^ Such assessment is based on three levels of severity (the higher the level, the more severe the symptoms) and is cluster-specific, meaning that DSC and RRB symptoms are rated separately (American Psychiatric Association, 2013, pp. 51–52). In this sense, ASD configures as a bidimensional spectrum.

Notably, two other diagnostic criteria must be met for a diagnosis of ASD to occur, which respectively involve the onset of the symptoms and their impact on an individual’s social functioning (American Psychiatric Association, 2013, pp. 50, emphasis added):

C. Symptoms must be present in the early developmental period (but may not become fully manifest until social demands exceed limited capacities, or may be masked by learned strategies in later life).

D. Symptoms cause *clinically significant impairment* in social, occupational, or other important areas of current functioning.

It is worth noting that DSM-5 is not completely clear about what degree of variability is acceptable within the ASD category. For instance, although it is ultimately meant that all symptoms of cluster A must be present, from the text alone is unclear how many symptoms from points 1, 2, and 3 of Cluster A must be present for an ASD diagnosis to apply. For many disorders, the DSM specifies a number of symptoms from a list that are required for an individual to get diagnosed with such disorder (like in the case of Criterion B of ASD, see above). In other cases, the Manual tells us that *all* the symptoms from a list are to be observed (see the case of Criterion A of SPCD below). However, in the case of Criterion A of ASD, there is no specific indication on that matter.

Another potential source of confusion connects to the assessment of symptoms severity (see above), which according to DSM-5 can fall below Level 1 (American Psychiatric Association, 2013, p. 51). What is unclear is whether “below Level 1” is to be intended as ‘not clinically significant symptoms’ or rather as ‘absence of symptoms’, for instance. In either case, the possibility that symptoms severity can fall below Level 1 (without ruling out a diagnosis of ASD) implies that an individual could, in principle, be diagnosed with ASD even if she has no (or has very mild) symptoms from Cluster A, and thus has only symptoms from Cluster B, or vice versa.

However, this possibility is inconsistent with Criterion D of ASD, according to which “symptoms cause *clinically significant impairment* in social, occupational, or other important areas of current functioning” (American Psychiatry Association, 2013, p. 50, emphasis added). This also seems inconsistent with the DSM-5’s claim that “Autism spectrum disorder is diagnosed *only when* the characteristic deficits of social communication are accompanied by excessively repetitive behaviors, restricted interests, and insistence on sameness. […] In addition to the social communication deficits, the diagnosis of autism spectrum disorder requires the presence of restricted, repetitive patterns of behavior, interests, or activities” (American Psychiatric Association, 2013, p. 31, emphasis added; see also p. 53).

Answering these questions one way or another would arguably make much difference in terms of diagnosis and intervention. Relatedly, admitting that the severity of symptoms from one cluster can fall below Level 1 seems inconsistent with the DSM-5 revisions in the categories of Communication Disorders (CDs), which we will discuss in the next section.

## Nosological revisions from DSM-IV and DSM-5: communication disorders

The DSM macro-category of Communication Disorders (CDs) includes a variety of disorders that are often dubbed Specific Language Impairments (SLI), a label that was never officially included in the DSM but that is largely used by researchers to indicate language difficulties in the absence of other neurodevelopmental deficits. In DSM-5, CDs include, for instance, Language Disorder, Speech Sound Disorder, and Childhood-Onset Fluency Disorder.

Usually, individuals affected by SLI have mild to severe difficulties in the comprehension and/or production of vocabulary, sentence structure, and discourse, not due to other medical or neurological reasons, nor to other neurodevelopmental disorders.

In the past, some authors have proposed that a further category was to be added to CDs to cover individuals who show impairments in linguistic communicative performance, but not in phonological and structural linguistic competence: that is, a pragmatic language impairment category, often dubbed PLI in research contexts (Adams & Bishop, 1989; Bishop & Rosenbloom, 1987; Rapin & Allen, 1983). Even if distinguishing children with SLI from those with PLI on pragmatic language tasks may be difficult (Ketelaars & Embrechts, 2017; Leyfer et al., 2008), the new DSM-5 disorder of SPCD can be seen as bridging the above nosological gap. In this view, SPCD is meant to pick out those language and communication difficulties that do not fall within the range of typical SLI, as individuals with SPCD may have normal phonological processing, vocabulary, and higher-order grammatical and semantic skills (Freed et al., 2010; Ryder et al., 2008; Taylor & Whitehouse, 2016).

In DSM-5, SPCD is characterized by the following diagnostic criteria (American Psychiatric Association, 2013, pp. 47–48):

A. Persistent difficulties in the social use of verbal and nonverbal communication as manifested by *all* of the following (emphasis added):

1. Deficits in using communication for social purposes, such as greeting and sharing information, in a manner that is appropriate for the social context.
2. Impairment of the ability to change communication to match context or the needs of the listener, such as speaking differently in a classroom than on a playground, talking differently to a child than to an adult, and avoiding use of overly formal language.
3. Difficulties following rules for conversation and storytelling, such as taking turns in conversation, rephrasing when misunderstood, and knowing how to use verbal and nonverbal signals to regulate interaction.
4. Difficulties understanding what is not explicitly stated (e.g., making inferences) and nonliteral or ambiguous meanings of language (e.g., idioms, humor, metaphors, multiple meanings that depend on the context for interpretation).

As in the case of ASD (see Sect. 2), in SPCD two further criteria are included involving the onset of symptoms and their impact on social functioning:

B. The deficits result in functional limitations in effective communication, social participation, social relationships, academic achievement, or occupational performance, individually or in combination.

C. The onset of the symptoms is in the early developmental period (but deficits may not become fully manifest until social communication demands exceed limited capacities).

As we mentioned in the Introduction, it is very plausible that SPCD was not solely introduced to fill a nosological gap of the macro-category of CDs. Rather, the choice of introducing this new disorder has connections with the revisions made to Autistic disorder and to the macro-category of PDDs more generally. In particular, SPCD seems to be also meant to account for subjects who would have had a DSM-IV autism-related diagnosis but do not now meet a DSM-5 diagnosis of ASD because they show DSC symptoms but no RRB ones (currently or by history) (Brukner-Wertman et al., 2016; Flax et al., 2019; Gibson et al., 2013; Regier et al., 2013; Swineford et al., 2014).

To clarify this, let us compare the whole list of symptoms and diagnostic criteria of ASD and SPCD.

On the one hand, ASD requires that (1) both DSC and RRB symptoms must be present (currently or by history), (2) all three kinds of DSC symptoms must be displayed (but this is never clearly stated, see above); (3) two out of four kinds of RRB symptoms must be displayed, and (4) both DSC and RRB symptoms must cause clinically significant impairment to the subject.

On the other hand, SPCD requires that (1) only DSC symptoms must be present, (2) symptoms from all four kinds of DSC symptoms must be present, and (3) RRB symptoms must have never manifested, either currently or by history.

One aspect that leaps out is that the SPCD symptoms are quite similar to the symptoms included in Cluster A of ASD, namely, deficits in social communication and social interactions (DSC). However, SPCD involves no RRB symptoms. So, the main difference-maker between the two disorders appears to be the presence of RRB symptoms (regardless of whether such symptoms are currently present or by history). In order to make a diagnosis of SPCD, it is in fact essential to first rule out a diagnosis of ASD by verifying the absence of RRB, either currently or by history.^8^

An implication of this is that, through this revision, the manual accepted the possibility that two individuals with very similar or nearly identical symptoms from the DSC domain will be diagnosed differently—one with ASD and one with SPCD. This can happen whereby both individuals show only DSC symptoms but the first one had also symptoms from the RRB domain in the past, while the second one has never manifested such kind of symptoms (Swineford et al., 2014). Thus, it may be difficult to distinguish ASD and SPCD on the basis of behavioral profile alone (Reisinger et al., 2011).^9^

Another aspect that is worth stressing is that the description of SPCD does not explicitly demand that DSC symptoms cause clinically significant impairment. Rather, Criterion B of SPCD only requires that the deficits result in some sort of functional limitations in communication and social interactions. This seems to imply that symptoms can be at a sub-clinical level.^10^

At this point of the discussion, one might believe that, given the changes in the diagnostic criteria of autism and related disorders, and the gap in the class of language impairments, introducing SPCD as an independent category is logically justified. However, many scholars and practitioners have pointed out that nosological changes of this magnitude require careful consideration for both epistemological and pragmatical reasons (Cooper, 2015; Solomon, 2017a, 2017b).

In the remainder of the paper, we shall review current discussions on the validity and reliability of SPCD, particularly its independence from ASD.

## The quest for validators

Roughly speaking, scientific validators are kinds of evidence that can help decide whether a certain condition is a disorder, rather than a cluster of loosely-related signs and symptoms. In this sense, a given psychiatric category should be able to group together individuals characterized by a given pathology and not just superficially similar individuals. So, the available data and evidence can act as ‘normative standards’ for categorizing certain conditions as disorders or not and to maximize the validity and reliability of the DSM’s categories.

As the manual reports in the Introduction, a variety of indicators were considered for altering the structure of DSM-5, including “shared neural substrates, family traits, genetic risk factors, specific environmental risk factors, biomarkers, temperamental antecedents, abnormalities of emotional or cognitive processing, symptom similarity, course of illness, high comorbidity, and shared treatment response” (American Psychiatric Association, 2013, p. 12).^11^

Validity issues are at the core of DSM-5 revisions of, for instance, autistic and psychotic disorders, where the assessment of a variety of subtypes has been replaced by a dimensional approach aimed at rating the severity of a disorder’s core symptoms (on ASD, see Sect. 2; see also Sect. 5.2 below). Empirical evidence from epidemiology or neurobiology can also suggest lumping two different disorders into one category or splitting one category into two—for instance, in DSM-5, insomnia disorders and narcolepsy involved such two strategies, respectively.^12^

In this view, if two clusters of symptoms turn out to have different etiological paths at the genetic or neural level, for instance, then it may be that the two clusters are related to two different disorders. For instance, if SPCD turned out to have a completely different etiology from both ASD and other communication disorders, that would be evidence that SPCD is a separate disorder and thus requires a dedicated category within the psychiatry nosology.

However, it is unlikely that the study of etiology—nor of any other validator alone—is capable of drawing clear-cut distinctions between mere clusters of symptoms and mental disorders, or even between two distinct disorders, though similar. This can depend on a variety of aspects, including current limitations in biological sciences (Taylor et al., 2013; Vernes et al., 2008), but also the existent individual variability in the manifestation of symptoms as well as on conceptual aspects involved in the definition of mental disorders.

Thus, in DSM-5, it is recognized that “the current diagnostic criteria for any single disorder will not necessarily identify a homogeneous group of patients who can be characterized reliably with all of these validators. Available evidence shows that these validators cross existing diagnostic boundaries but tend to congregate more frequently within and across adjacent DSM-5 chapter groups” (American Psychiatric Association, 2013, p. 20). This means that various considerations are to be taken into account when we assess the promotion or the elimination of specific diagnostic categories—including etiological, pragmatic, social, and conceptual ones (Solomon, 2017a; Tabb, 2019; Zachar, 2015).

In the next sections, we will focus on three major, potential validators of SPCD, i.e., etiology, response to treatment, and measurability, and ask whether research on these aspects support the DSM-5 nosological changes outlined in Sects. 2 and 3.

### Etiology

The first aspect that we will be considering is whether the data from biological sciences about the etiology of ASD and SPCD can validate SPCD and support the introduction of SPCD as an independent nosological category in the DSM.

As we mentioned above, the etiology of mental disorders can involve a variety of aspects or ‘levels’ of the biological organization, including genetics, epigenetics, biochemistry, and neurobiology, but can also depend on environmental conditions and life events (Kendler, 2012; Murphy, 2006).

As regards autism-related disorders, it has been classically assumed that deficits in social interactions, deficits in communication, and restricted and repetitive behaviors—that is, the three main classes of symptoms of Autistic Disorder—constitute together some sort of “autism phenotype”, sometimes called Broader Autism Phenotype or BAP (see, e.g., Georgiades et al., 2013; Piven, 2001; Piven et al., 1997; Sucksmith et al., 2011), and were connected by a common genetic etiology (Bailey et al., 1995; Constantino et al., 2004, 2006; Folstein & Rutter, 1977; Piven & Folstein, 1994; Rutter, 2000; Wing & Gould, 1979). Moreover, the BAP was generally assumed to be common to clinical and subclinical populations displaying differing levels of RRB and socio-pragmatic impairments (Bailey et al., 1998; Constantino et al., 2006; Sucksmith et al., 2011).

Before the publication of DSM-5, however, the BAP assumption was challenged by various findings showing that people that exhibit severe deficits in social interactions tend to exhibit also severe deficits in communication, but do not necessarily exhibit equally severe symptoms with regard to restricted and repetitive behaviors, and vice versa (Dworzynski et al., 2009; Mandy & Skuse, 2008; Mandy et al., 2011; Pooni et al., 2012). Other studies argued instead for the independence of the three classes of symptoms—that is, deficits in social interactions, communication, and rigid/repetitive traits—at the genetic, cognitive, and neural levels (Happé & Ronald, 2008; Happe et al., 2006; Ronald et al., 2006, 2011).^13^

In general, as impairments in social interactions and in communication appeared to correlate stronger with each other, it was somewhat easier to assume that they had a common etiology, so they were categorized into the single domain of DSC. By contrast, as symptoms from the DSC domain appear not to strictly correlate with symptoms from the RRB domain, different etiologies and causal pathways for the two domains were suggested. However, the quest for two distinctive causal pathways, consistently associated with the two different clusters of symptoms, has been unsuccessful.

Many genetics studies published before the introduction of the new categories of ASD and SPCD already converged on the conclusion that a common etiology for DSC and RRB is likely. As reported by Taylor and Whitehouse in a recent review (2016), genetic variants were found in common between Autistic Disorder and some CDs (Alarcon et al., 2008; Arking et al., 2008; Bakkaloglu et al., 2008), and between both of them and pragmatic impairments (St Pourcain et al., 2013).

Studies on the category of SPCD are not numerous, but the hypothesis that SPCD and ASD have different etiologies does not seem to have solid bases here either. For instance, Flax and colleagues (2019), analyzed the data collected from a large number of families that included subjects diagnosed with ASD, subjects with structural language impairments, and healthy individuals. The authors looked for evidence of the presence of social communication issues in the absence of structural language impairments and of clinical levels of RRBs. Results from all three groups showed that there exists a positive correlation between RRBs and pragmatic language impairments, suggesting that the SPCD category does not represent an independent condition.

Further indirect evidence against the independence of RRB and DSC symptoms was provided, for instance, by Brukner-Wertman and colleagues (2016). The authors noted that, if the two etiologies were independent of each other, then it would be plausible expecting to observe not only people with DSC without RRB (as in the case of SPCD), but also people with RRB without DSC. The fact that people with this symptomatic profile have not been found^14^ may suggest that the DSC and RRB domains are not totally independent.

Although the results above are certainly not definitive, the existing data do not firmly support the hypothesis that SPCD has a specific etiology. In Sect. 6, we will return to the potential role of SPCD as a research entity and how this could possibly enhance our understanding of its etiology. In the next section, instead, we shall consider alternative sources of evidence for the validation of SPCD.

### Response to treatment

Especially when etiologies are highly speculative or the data unclear, as in the case at issue, elements other than etiology can be taken into account for assessing the promotion or the elimination of specific diagnostic categories (Tabb, 2019; Zachar, 2015). In this section, we shall assess a second class of potential validators of the SPCD diagnostic category, namely, how patients diagnosed with the disorder tend to respond to treatment.

According to some scholars, treatments that proved to be successful for autism-related disorders or SLI are poorly effective on people with pragmatic impairments (Gerber et al., 2012), who instead do successfully respond to treatments that are specifically tailored for them, such as the Social Communication Intervention, an individualized intervention that targets social understanding and interaction, verbal and nonverbal pragmatic skills, and language processing (Adams & Gaile, 2015; Adams et al., 2015; Adams et al., 2020; Adams et al., 2012; Adams et al., 2011; Gaile & Adams, 2018). This might suggest that SPCD is an independent nosological category.

However, a conceptual point is worth making as to what response to treatment can really tell us on the validity of mental disorders (and pathologies more generally).

Generally speaking, from the fact that people from one group of the clinical population respond positively to a given treatment, and people from another do not, it does not necessarily follow that people from the two groups suffer from different diseases. For instance, it well may be that they suffer from the same disease with different severity levels. So, it is possible that mild and severe forms of the same disease require different treatments—let us think, for example, of the simple case of a severe migraine compared to a mild one. Similar reasoning might apply to mental disorders like SPCD, too. For the sake of the argument, let us assume that people diagnosed with SPCD poorly respond to treatments that are effective to people diagnosed with ASD or SLI and, vice versa, they successfully respond to treatments that are otherwise ineffective to people diagnosed with ASD or SLI. This, however, does not suffice for considering SPCD an independent nosological category; it may well be, for instance, that SPCD is a sub-class or a milder form of ASD (we will return on this possibility in Sect. 6).

Unfortunately, the available evidence of different responses to treatment is insufficient for settling the issue. First, a common problem of studies on therapies targeted on social communication impairments is that they usually do not exclude ASD patients (who also often have communication impairments). So, most available data cannot be taken to be specifically about SPCD, but rather involve heterogeneous groups of subjects (Topal et al., 2018).

Second, one may wonder whether all individuals with SPCD would equally benefit from a standardized treatment (Gerber et al., 2012) or would have similar reactions to the same intervention; this might cast doubts on the idea that there would be any actual difference in the response to treatment between SPCD and ASD. Relatedly, one might also ask whether interventions targeting only social-communicative skills would overlook other relevant deficits shared by individuals diagnosed with SPCD—for some examples, see Brukner-Wertman et al. (2016).

### Measurability

Let us now consider a third element that can be considered to assess the validity of a new diagnostic category like SPCD, namely, its measurability. In this context, the question of measurability concerns whether a psychological trait or disease, like SPCD, can be reliably measured and assessed independently from other psychological constructs or diseases.

As a general strategy, a way to assess the validity of SPCD would be to determine whether there are *specific* deficits in social communication that appear among the SPCD symptoms but that do not appear in the description of ASD or of other relevant communication disorders. *Prima facie*, looking at the DSM-5 diagnostic criteria of ASD and SPCD, it would seem so.

As we outlined in Sects. 2 and 3, ASD symptoms include deficits in social-emotional reciprocity, deficits in nonverbal communicative behaviors, and deficits in developing, maintaining, and understanding relationships. By contrast, SPCD symptoms appear to be more ‘fine-grained’ and language-related, and include deficits in using communication for social purposes, deficits in the ability to change communication to match context or the needs of the listener, deficits in following rules for conversation and storytelling, deficits in understanding what is not explicitly stated and nonliteral or ambiguous meanings of language.

Even if symptoms are described in a different jargon and focus on slightly different aspects of social and pragmatic behavior, the existing literature on SPCD highlights some lack of specificity in the diagnostic tools targeting the pragmatic component of communication that would characterize SPCD symptoms (Flax et al., 2019; Gerber et al., 2012; Ketelaars & Embrechts, 2017; Topal et al., 2018). However, other existing tools might be used to measure the diagnostic features of SPCD (Norbury, 2014; Yuan & Dollaghan, 2018).

Notably, social communication and pragmatic abilities are difficult to measure in standardized ways as they are a set of contextually dependent human behaviors that occur in dyadic exchanges (Adams, 2002; Volden et al., 2009) and are highly subjected to cultural variations (Carter et al., 2005).

The most widely used test is the Children’s Communication Checklist-2 (CCC-2), which addresses structural language, higher-order language, pragmatics, social abilities, and interests (Bishop, 2003a, 2013) and is one of the few norm-referenced and validated questionnaires to measure pragmatic deficits.^15^ The CCC-2, combined with the Social Interaction Deviance Composite (SIDC) (Bishop, 2003a, 2003b), is in fact able to identify pragmatic abilities that are disproportionately impaired relative to structural language competencies. Unfortunately, the results of the tests may vary considerably depending on the person who completes the questionnaire (e.g., the parents or the teachers) and on the specific context of interaction (Bishop & Baird, 2001). To avoid this problem Bishop and colleagues have formulated a self-reported test, the CC-self-report (Bishop et al., 2009), but it is suitable for older children only and it is unclear whether it adequately takes into account the issue of the lack of self-awareness, that can easily compromise the reliability of the results (Ketelaars & Embrechts, 2017). Leaving these problems aside, it has been pointed out that the CCC-2 cannot screen off children who have SLI or ASD, which are exclusionary diagnoses for SPCD, as the test scores are continuously distributed with no clear categorical boundaries (Flax et al., 2019; Laws & Bishop, 2004; Norbury et al., 2004).

Regardless, from a conceptual point of view, it should be noted that measurability can unlikely provide bulletproof justifications of the validity of a nosological category. From the fact that a behavioral or psychological construct can be measured or even operationalized, it does not follow that it is an independent disorder or category. For example, pain can be measured, and it is considered a valid construct or research entity for the sake of research on painkillers that can be targeted by pharmacological studies; but this does not make it a nosological category in either somatic medicine or psychiatry.^16^

## Reliability and nosological inconsistencies

In this section, we shall consider three sorts of inconsistencies arising from the DSM-5 revision regarding ASD and SPCD. It should be noted that any change in psychiatric nosology can bring with it some practical shortcomings for patients, such as losing eligibility for clinical services, healthcare assistance, and social support (Cooper, 2015). For this reason, a cautionary approach to nosological revision is generally recommended, as it can impact people’s lives in many (often unexpected) ways.

In the case of ASD, we will explain that even additional caution is appropriate due to three peculiarities of the nosological revision: first, the introduction of the so-called “grandfather clause”, which generates contradictions between the two diagnostic systems of DSM-IV and DSM-5 (Sect. 5.1); second, the introduction of the concept of spectrum in place of the concept of category, which implies the coexistence of two different (and arguably incompatible) views of ASD, i.e., a dimensional and a categorical one (Sect. 5.2); and third, the introduction of thresholds in the definition of autism disorders, which opens up a variety of questions about how to draw a line between clinical and sub-clinical levels of symptoms severity (Sect. 5.3).

### Contradictions in diagnoses and the grandfather clause

Many scholars and patients’ advocates have expressed their worries about the practical consequences of the DSM-5 revisions relating to ASD (Autistic Self Advocacy Network (ASAN), 2012a, 2012b; Greenberg, 2013, pp. 296–299; Ne’eman & Kapp, 2012). Indeed, as we mentioned above, losing an ASD diagnosis would imply the exclusion from a well-established network of organizations engaged in clinical services, healthcare assistance, education, employment, economic support, and research.^17^ To mitigate worries of this sort, the DSM-5 Task Force included the following clause in the ASD diagnosis:

“Individuals with a well-established DSM-IV diagnosis of autistic disorder, Asperger’s disorder, or pervasive developmental disorder not otherwise specified [PDD-NOS] should be given the diagnosis of autism spectrum disorder. Individuals who have marked deficits in social communication, but whose symptoms do not otherwise meet criteria for autism spectrum disorder, should be evaluated for social (pragmatic) communication disorder” (American Psychiatric Association, 2013, p. 51).

As a consequence of this exemption, which is sometimes dubbed “grandfather clause,” someone who had already been diagnosed with DSM-IV Autistic Disorder, Asperger’s Disorder, or PDD-NOS, should be automatically given a diagnosis of ASD after the publication of DSM-5.

Interestingly, such an ASD diagnosis would be ‘inherited’ even if the current symptomatology would suggest a different diagnosis or even no diagnosis at all under the DSM-5 diagnostic criteria. For instance, an individual who had been diagnosed with PDD-NOS before 2013, and who would now meet the diagnostic criteria for SPCD, would be diagnosed with ASD thanks to the grandfather clause. Likewise, an individual that would now have no diagnosis at all (because she meets neither the ASD criteria nor the SPCD ones), would still be diagnosed with ASD if she had a DSM-IV diagnosis of Asperger’s Disorder or PDD-NOS, for instance.

Consequently, individuals who currently have the same symptoms and needs might be given different diagnoses or no diagnosis at all, with important clinical and practical consequences. For instance, someone who received a PDD-NOS diagnosis before 2013, and thus inherited an ASD diagnosis because of the grandfather clause, could access a variety of healthcare services relating to autism diagnoses; but, after the publication of DSM-5, an individual with very similar symptoms and needs would be excluded from such services if a diagnosis of SPCD was made instead (Bishop, 2010; Dockrell et al., 2012). It is worth noting that this is not just an abstract possibility as it has been shown that many individuals that were diagnosed with DSM-IV PDD-NOS may currently be eligible for a DSM-5 SPCD diagnosis (Kim et al., 2014; Regier et al., 2013).

More generally, one problematic aspect of the grandfather clause is that it extends the lifespan of some DSM-IV categories and thus creates two competing—and to a certain extent contradictory—diagnostic systems, both of which are currently in use (Smith et al., 2015, p. 2542). Such a situation is quite unique and certainly odd if compared to other psychiatric categories.

The oddness is even greater when another footnote is made explicit. As Cooper (2015) points out, the DSM-IV put an ‘or’ instead of an ‘and’ among the diagnostic criteria of PDD-NOS due to a copyediting error (an error that was corrected later in DSM-IV-TR).^18^ Hence, it was theoretically possible to diagnose someone with PDD-NOS in the absence of problems with social interaction (First & Pincus, 2002), thus diagnosing an individual whose sole symptom was stereotyped behavior, interests, and activities. This means that the grandfather clause did not only ‘fossilize’ an old diagnosis, but also an unintentionally mistaken one (Cooper, 2015, p. 47).

One may object to this line of argument that the DSM is a pragmatic tool that tries to accommodate heterogeneous and competing interests from the many different actors involved, such as clinicians, scientists, pharmaceutical and insurance companies, patients and their families (on these general aspects, see Cooper, 2015; Pickersgill, 2012; Solomon, 2017a); in this view, the presence of contradictions—such as those generated by the grandfather clause—come as no surprise.

However, contradictions in diagnosis should not be taken lightly, even if they are difficult to avoid. The DSM-5 is a “living document” (American Psychiatric Association, 2013, p. 13) and must be able to evolve in the light of scientific discoveries and conceptual criticisms. This is especially pressing since the DSM has the power to define who is mentally disordered and who is not, with all its moral and practical consequences. Even more important is the fact that contradictions of the sort above are by no means inevitable, as we shall discuss in Sect. 6.

### Nosological redundancy and the concept of spectrum

One of the reasons for introducing the category of Autism Spectrum Disorder—and thus merging together the DSM-IV diagnoses of Autistic Disorder, Childhood Disintegrative Disorder, Asperger’s disorder, and PDD-NOS—was the widespread intuition that autism should be regarded as a spectrum, rather than a group of disorders categorically distinct from each other (Chakrabarti et al., 2009; Lundstrom et al., 2012; Wing & Gould, 1979; see also Sect. 2).

Indeed, the manifestation of autistic symptoms varies greatly depending on factors such as the severity of the autistic condition, the developmental level, and the chronological age. Thus, ASD is meant to encompass a variety of conditions characterized by varying levels of symptoms severity—ranging from low (or even zero, see Sect. 2) to severe—that were previously considered as independent disorders, such as Early Infantile Autism, Childhood Autism, Kanner’s Autism, High-functioning Autism, Atypical Autism, Childhood Disintegrative Disorder, Asperger’s Disorder, and PSS-NOS (American Psychiatric Association, 2013, p. 53).^19^

The introduction of a spectrum, however, brought about a variety of inconsistencies, particularly as regards the comparison and differential diagnosis of ASD and SPCD and the actual range of applicability of the SPCD category. In what follows, we shall consider two alternative readings of SPCD—dimensional and categorical, respectively. Interestingly, such two interpretations are both supported by the DSM-5 characterization of SPCD, though they both come with peculiar issues and leave open conceptual as well as practical questions.^20^ But let us proceed step by step.

Conceiving of DSM-IV autistic related disorders as a spectrum means, in the DSM-5’s own words, that “symptoms of these disorders represent a single continuum of mild to severe impairments in the two domains of social communication and restrictive repetitive behaviors/interests rather than being distinct disorders” (American Psychiatric Association, 2013, p. XIII). As seen in Sect. 2, ASD is here conceptualized within a bidimensional framework where each dimension represents one cluster of symptoms, namely, DSC and RRB (see Sect. 2).

**Fig. 1:**
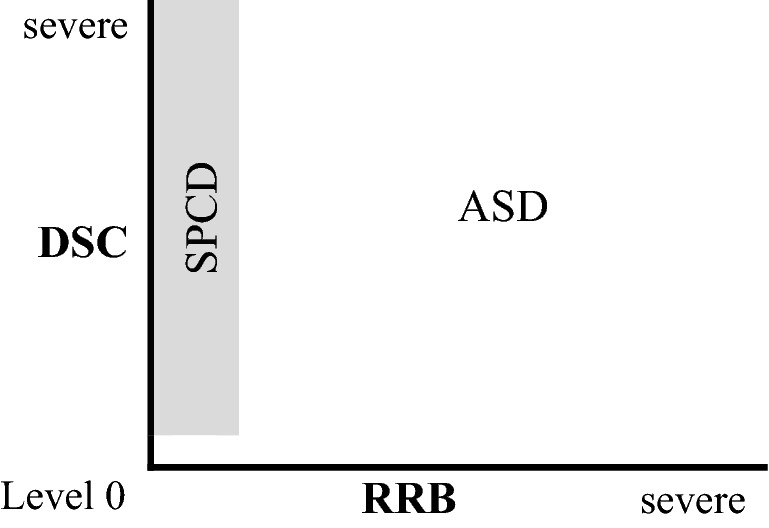
SPCD as a part of the spectrum of ASD

If we consider such two main classes of symptoms of ASD, one striking aspect is that an individual with low severity levels (or zero) of RRB symptoms will look very much like an individual with SPCD. So, in principle, a diagnosis of ASD could suffice to include individuals with symptoms from the DSC cluster only, because the spectrum of symptoms of ASD naturally includes the symptoms that are typically observed in SPCD patients (see Fig. 1). In this sense, SPCD, intended as an independent nosological category, appears to be redundant.

This problem was identified, for instance, by Mandy and colleagues (2017), who could not find evidence that SPCD is qualitatively distinct from ASD (see also Weismer et al., 2021). Rather, SPCD individuals appear to lie “on the borderlands of the autism spectrum” (Mandy et al., 2017, p. 1) or “between ASD and non-ASD” (Mandy et al., 2017, p. 8), describing people with autistic traits that do not meet the full criteria for an ASD diagnosis.

An alternative reading invites a categorical (rather than dimensional) interpretation of the DSM-5’s diagnostic criteria. To clarify this, let us focus on the requirements that RRB symptoms *must be present* currently or by history as well as that *they must cause clinically significant impairment* to the subject. These requirements suggest that ASD could be regarded as a disorder characterized by two specific diagnostic criteria that are individually necessary and jointly sufficient (it is in this sense that the disorder would be characterized in categorical terms, rather than dimensional). Such criteria involve the presence of both two clusters of symptoms of DSC and RRB, but also the fact that such symptoms must cause clinically significant impairment to the subject and must be present early in childhood. If this ‘categorical’ interpretation of ASD holds, then the DSM-5 diagnostic criteria do not really allow for low levels of symptoms expression or even their complete absence, as only symptoms ranging from a certain clinically significant threshold to severe, which are present currently or by history, can figure in an ASD diagnosis. If so, ASD and SPCD would come to appear as separate categorical entities.

Importantly, this categorical view could alleviate the problem above regarding the redundancy of SPCD, as the requirements could prevent the overlap of the two classes of symptoms, because a diagnosis of SPCD will apply only to those cases where RRB symptoms have *never been observed* (neither currently, nor by history). Such view, however, is clearly in contrast with the view of autism-related disorders as a spectrum where the value of one of the two dimensions of DSC and RRB can be below Level 1 (see Sect. 2).

Which of the two interpretations does better account for the aims of the DSM fifth version? Is there any way out of this inconsistency?

Although this aspect is never explicitly discussed in DSM-5, it seems to us that the inconsistency could be resolved by assuming that neither DSC nor RRB levels of symptoms severity can have values below a certain threshold (e.g., below Level 1). This possibility is somehow reflected by Criterion D of ASD, according to which symptoms should cause clinically significant impairment in social, occupational, or other important areas of current functioning (see Sect. 2)—if we take ‘clinically significant’ as meaning ‘above a clinical threshold’, of course. If so, the class of individuals with SPCD would not overlap with the class of people with ASD because people with SPCD would always have RRB symptoms severity equal to zero, while people with ASD would always have RRB symptoms beyond the threshold of clinical significance.

Although this may look like a reasonable solution, with the introduction of thresholds more problems come along. In the next section, we shall focus on this aspect.

### Clinical thresholds and nosological grey areas

In DSM-5, thresholds are understood as points where the experience associated with one symptom (or a group of symptoms) becomes problematic or pathological. Notably, thresholds are considered as culturally- and context-sensitive, since defining what is problematic or abnormal “depends on cultural norms that are internalized by the individual and applied by others around them, including family members and clinicians” (American Psychiatric Association, 2013, p. 14). This is the case for ASD, too (Mandy et al., 2014). In this view, the DSM-5’s distinction between mental health and pathology involves conventional or pragmatic elements and can revolve around various parameters, including the number of symptoms, their clinical significance, or their duration (Jang, 2005; Knopik et al., 2017).

One of the main reasons for introducing the concept of clinical threshold in the psychiatry nosology is surely the attempt of reducing the occurrence of overdiagnosis and overtreatment, which bring with them the undesirable consequences of labeling as ‘disordered’ people who merely behave ‘oddly’, for instance, with a variety of negative consequences, e.g., stigmatizing individuals and increasing the costs of medical support.^21^

Unfortunately, in the case of ASD and SPCD, appealing to thresholds has some problematic consequences, particularly the introduction into the Manual of some ‘grey areas’ where there is no clear answer to whether an individual is to be diagnosed in a way or another, or not diagnosed at all.

For instance, let us consider someone who has DSC symptoms but sub-clinical RRB symptoms (namely, the severity level of RRB symptoms is below the clinical threshold, however the threshold is defined or assessed). In this case, psychiatrists may disagree as to what diagnosis is better suited for the subject, if any. On the one hand, a diagnosis of SPCD could conflate with the principle that “a diagnosis of social (pragmatic) communication disorder should be considered only if the developmental history fails to reveal any evidence of restricted/repetitive patterns of behavior, interests, or activities” (American Psychiatric Association, 2013, p. 49). Indeed, although RRB symptoms may not reach the clinical threshold, *they may still be present*, and this could discourage a SPCD diagnosis. On the other hand, a diagnosis of ASD would conflate with Criterion B of ASD, according to which *at least two out of four* RRB symptoms should be present at a clinically significant level, currently or by history (see Sect. 2).

So, an individual with DSC symptoms but *sub-clinical RRB symptoms* or just *one out of four RRB symptoms* could receive no diagnosis. It is worth noting that people affected by DSC and stereotyped language—which is now considered as one variety of RRB (see Sect. 2)—are quite common, as are individuals affected by DSC and subclinical RRB symptoms (Swineford et al., 2014). In fact, as Nordbury (2014, p. 211) points out, “It is likely that even if children with SPCD do not exhibit enough [RRB]s to meet threshold for ASD, they have elevated levels of [RRB]s relative to peers”. However, given the current diagnostic criteria, they would be diagnosed neither with ASD nor SPCD. This delineates a sort of ‘gap’ in the diagnostic system of DSM-5.

A second issue regards the fact that, although clinical thresholds appear to be mentioned in the description of ASD (see Sect. 5.2), they are definitely not considered in SPCD. So, a diagnosis of ASD applies only when DSC and RRB symptoms are clinically significant, thus avoiding overdiagnosis and overtreatment. But what about individuals with sub-clinical DSC symptoms and no RRB symptoms, then? At the present state, DSM-5 would in principle allow one to diagnose such individuals with SPCD even if they exhibit just odd behaviors and do not necessitate any specific treatment. Unless some threshold is introduced in the SPCD category too, this may push forward the risk of overdiagnosis and medicalization of healthy individuals.

Finally, it should be noted that the concept of threshold itself is barely defined even in more basic science like genetics and developmental biology (Serpico, 2020).^22^ This brings about some arbitrariness into diagnosis as to what ‘sub-clinical’ means, decreasing the DSM-5’s reliability. For instance, in the case of RRB, some psychiatrists may understand it as the complete absence of RRB, while others may understand it as having some form of RRB, though ‘not problematic’—whatever this means.

It is not the aim of this paper to investigate the concept of threshold and delineate a distinction between clinical and sub-clinical symptoms in ASD. But we would like to highlight that the introduction of thresholds would pave the way to a variety of ambiguities as regards the status of SPCD and ASD. This is a cost that might not be worth paying—certainly not if the very purpose of the concept of threshold is justifying the introduction of SPCD as an independent disorder in the DSM.

## Concluding discussion on the future of SPCD

In this paper, we reviewed the existing literature on the validity and reliability of the DSM-5 category of Social Pragmatic Communication Disorders (SPCD) and discussed epistemological and ethical questions raised by the DSM-5 revision of Pervasive Developmental Disorders (PDDs) and Communication Disorders (CDs).

In the first part, we summarized the major aspects of this nosological revision, particularly the introduction of the categories of Autism Spectrum Disorder (ASD) and SPCD.

Then, we reviewed the literature on SPCD as regards three major validators, namely, etiology, response to treatment, and measurability. A general message arising from the material here reviewed is that, at the current state of evidence, the cluster of symptoms associated with SPCD appears not to be independent of the cluster of symptoms associated with ASD.

Finally, we pointed to three types of inconsistencies in the DSM contemporary nosology that originate from the DSM-5 revision of PDDs and CDs, some of which are due to the existence of two competing diagnostic systems (from DSM-IV and DSM-5, respectively), some others to the appeal to the concepts of *spectrum* and *threshold*, which both generate diagnostic ‘grey areas’ or redundancy. Taken together, these three key issues cast doubts on the actual reliability of diagnoses in the DSC and RRB domains.

In the final part of the paper, we also explained that a decision is yet to be made between two possible interpretations of the status of ASD that is, as a dimensional or a categorical disorder. Notably, both such alternatives are somehow compatible with the available scientific evidence, but none of them is at present fully endorsed by DSM-5; and still, both have different implications for the inclusion or exclusion of SPCD from the psychiatry nosography.

First, we can consider ASD as a bidimensional spectrum where the symptoms can range from zero to severe; this would lead us to delete the SPCD nosological category as redundant, since individuals with DSC symptoms could be diagnosed with both SPCD and ASD (i.e., ASD without RRB symptoms would be phenotypically identical to individuals with SPCD). A second option is to consider ASD as a bidimensional spectrum but introduce a ‘threshold requirement’, thus denying that symptoms can be at a sub-clinical level for a diagnosis to occur. This option would allow us to consider ASD and SPCD as two independent disorders but would have unpalatable consequences in terms of clinical decision making relating to the definition of clinical and sub-clinical symptoms. This would decrease the reliability of both diagnoses.

In the remainder of the paper, we would like to consider whether SPCD could play any role in contemporary psychiatry other than that of an independent mental disorder. An interesting aspect is that the evidence supposedly in favor of SPCD as an independent category is also compatible with ‘more conservative’ options, none of which require us to revise the psychiatric nosology as drastically as DSM-5 did.^23^ We will be considering three main options that can be detected in the debate on SPCD: first, the reduction of SPCD to a subtype of another disorder or macro-category; second, the conceptualization of SPCD as a cluster of symptoms; and third, its conversion into a research entity. Let us see them one by one.

A first possibility is the introduction in the DSM of a subtype of one existing macro-category or disorder falling within CDs, SLI, or ASD. This proposal was made, for instance, by Jill Boucher who, in a 1998 clinical forum on the issue, concluded that “It appears that if SP[C]D is to be confirmed as a distinct diagnostic entity there are two possible niches which it could logically occupy: 1. It could be a subtype of autism; 2. It could be a subtype of SLI. There is, of course, a third possibility which builds on the other two; 3. That SP[C]D qualifies as a subtype of autism and as a subtype of SLI, forming what would be a most interesting intermediate disorder” (Boucher, 1998, p. 79). The view of SPCD symptomatology as part of a broader category is also supported by Helen Tager-Flusber (2018), who wrote: “There are no new assessment tools, no clearer diagnostic criteria, no stronger evidence for the existence of the condition and no innovative, effective interventions. This is not to say that pragmatic impairments don’t exist. On the contrary, they appear prominently as a core feature of autism and as a co-occurring condition for many children and adults with neurodevelopmental condition”.^24^

A second palatable option is considering SPCD as a special cluster of symptoms, including social communication and pragmatic language impairments, that are statistically associated with each other and thus tend to display and evolve together (Norbury, 2014). To make a comparison, let us think about symptoms such as cough, fever, and fatigue: although they often come together, it is well known that they are not indicative of any specific, independent somatic disease. Rather, they are part of a cluster of symptoms that is common to many somatic diseases, such as different kinds of viral influenza, pulmonary inflammation, and so on.

If we consider the comorbidity of SPCD with other mental and somatic conditions, the interpretation of SPCD as a cluster of symptoms sounds very plausible. Indeed, symptoms from the DSC domain are common to many different mental and somatic conditions, such as ADHD, William’s Syndrome, Conduct disorder, Closed head injury, and Spina bifida. This suggests that the presence of DSC symptoms without RRB symptoms is not necessarily indicative of a specific mental disorder, like SPCD.

That the signs and symptoms of a proposed diagnosis tend to cluster together is, of course, an empirical hypothesis that needs to be further tested. However, an intriguing aspect of this interpretation of SPCD is that, just like nosological categories, clusters of symptoms can provide the basis for pharmacological or clinical studies more generally. Unlike nosological categories, however, they do not have an immediate impact on healthcare and ultimately on the everyday management of patients.

This leads us to a third potential interpretation of SPCD, according to which SPCD is to be considered as a research entity.^25^ Within a research agenda on SPCD, one could choose to bet on the distinction between the DSC and RRB domains and investigate whether there actually exist two distinct etiologies (see Sect. 4.1). This could certainly be beneficial for biomedical research as it would allow us to further investigate the validity of SPCD; at the same time, taking SPCD as a research entity would not reshape the psychiatry nosology directly, which seems to us essential due to the unexpected effects this might have on the clinical practice.^26^ If so, more studies will be hopefully carried on, as some scholars recommended (Mandy et al., 2017; Topal et al., 2018).

Similar considerations were made, for instance, by Brukner-Wertman and colleagues (2016, p. 2823), who pointed out that if the etiologies of DSC and RRB are independent, then not just one, but two new nosological categories should be logically introduced: not only SPCD, to cover DSC symptoms without RRB, but also another category aimed at covering RRB symptoms without DSC.^27^ However, Brukner-Wertman and colleagues noticed that the implementation of a research hypothesis in a clinical manual could be dangerous in terms of the impact on many patients’ life.

This connects to a general question regarding what factors, other than scientific evidence, can guide the delineation of the future psychiatric nosology.

That both epistemic and non-epistemic factors can impact scientific taxonomies is a central topic in contemporary philosophy of science (Boyd, 1999; Chakravartty, 2007; Craver, 2009; Dupré, 1993; Hacking, 1991; Onishi & Serpico, 2021). In the case of psychiatric categories, this can involve different aims characterizing different sub-fields of psychiatry (e.g., explanation, treatment, prevention), but also normative judgments aimed at avoiding potentially negative social or clinical outcomes (Cooper, 2015; Solomon, 2017a; Tabb, 2019; Zachar, 2015).

For instance, an aspect that should be taken into account is that, if SPCD is regarded as an independent disorder belonging to the macro-category of CDs, then the range of treatment options accessible to individuals diagnosed with SPCD would be much limited. In principle, if therapies specifically designed for ASD existed, they might not even be considered for a child diagnosed with SPCD, even if the impairments in the socio-communicative domain may be the same. Even more worrisome is that, in practice, a child diagnosed with SPCD could not access therapies dedicated to subjects affected with ASD who can rely on a deep-rooted system of healthcare support (Autistic Self Advocacy Network (ASAN), 2012a, 2012b; Ne’eman & Kapp, 2012). It has already been documented not only that funding for research on ASD far exceeds that for CDs (Bishop, 2010), but also that children with ASD receive far more intensive and regular educational support (Dockrell et al., 2012; Tanguay, 2011; Weismer et al., 2021; see also Sect. 5.1 above). As Brukner-Wertman and colleagues noted, “Since the diagnosis of autism is associated with a well-established network of organizations engaged in public health, education, employment, economic benefits and research, excluding SPCD from this network raises the question of how will the official systems deal with it” (Brukner-Wertman et al., 2016, p. 2826). Relatedly, Weismer et al., (2021) argued that “children with ASD and SCD may have overlapping service needs and we may be overlooking concomitant psychopathology or subtle RBB manifestations in SCD cases if we focus solely on treatment of social communication” (﻿Weismer et al., 2021).

In the absence of conclusive empirical evidence, and in the light of the uncertainty outlined throughout the paper, it seems to us very reasonable that the pragmatic and ethical considerations above should enter the decision of introducing, maintaining, or eliminating a category like SPCD. More generally, we believe that assessing non-evidential reasons and background conceptual assumptions should be part and parcel of both the philosophical and the scientific work, especially in domains like psychiatry, where the consensus is arguably less firmly established than in other scientific endeavors.

## Footnotes

1 It is worth noting that while most PDDs have been integrated into ASD, others, like Rett syndrome, have been removed from the DSM and can now be found among Developmental Anomalies in the International Classification of Diseases (ICD). See https://icd.who.int/browse11/l-m/en#/http%3a%2f%2fid.who.int%2ficd%2fentity%2f201200685.

2 Relatedly, several studies identified subjects with pragmatic language impairments but no restricted and repetitive patterns of behavior, interests, or activities that are typical of autism-related disorders—a group of individuals sometimes called “children in question” (Adams & Bishop, 1989; Bishop & Adams, 1989; Botting & Conti-Ramsden, 1999).

3 See https://www.spectrumnews.org/opinion/viewpoint/narrowing-autism-dsm-5-runs-counter-idea-broad-spectrum.

4 As we shall explain shortly, such two clusters can be understood as two dimensions where symptoms can have different severity levels.

5 This decision is not free from objections, though; for a brief review see Norbury (2014).

6 Note that this parameter is specified outside the diagnostic criteria, in the Specifiers section. “The severity specifiers (see Table 2) may be used to describe succinctly the current symptomatology (which might fall below level 1), with the recognition that severity may vary by context and fluctuate over time. Severity of social communication difficulties and restricted, repetitive behaviors should be separately rated” (American Psychiatric Association, 2013, p. 51).

7 More generally, DSM-5 recognizes that the boundaries between many disorders are more fluid than DSM-IV acknowledged, and that it “should accommodate ways to introduce dimensional approaches to mental disorders, including dimensions that cut across current categories. Such an approach should permit a more accurate description of patient presentations and increase the validity of a diagnosis” (American Psychiatric Association, 2013, p. 5).

8 Here, we do not mean to underestimate the importance of RRB symptoms in the definition of ASD. However, it should be noted that some manifestations of ASD are in fact very similar to SPCD. In this sense, some scholars have expressed the worry that SPCD could be treated as a residual category for ‘not-quite’ ASD individuals (Skuse, 2012).

9 We will return to this problem in Sect. 5.2.

10 We will return to this aspect in Sect. 5.3.

11 For a more comprehensive theoretical framework of validation, see Kendler et al. (2009).

12 On lumping and splitting strategies in biology and biomedical sciences, see Craver (2009) and McKusick (1969).

13 It should be noted that if social communication and pragmatic language impairments do not necessarily go together, then the requirement that both clusters of symptoms must be present for a diagnosis of SPCD becomes questionable (Norbury, 2014).

14 One might note that symptomatology including RRB but in the absence of DSC characterizes Obsessive–Compulsive Disorder (OCD). In fact, some forms of RRB tend to shade into symptomatic profiles that are more typically associated with Obsessive Compulsive Disorder (OCD) (Leekam et al., 2011; Zandt et al., 2009). However, it should be noted that RRB is a very heterogenous category. If we look at the types of RRB symptoms that are typically associated with ASD and OCD, we can notice that such manifestations are quite different from each other. In OCD, repetitive behaviors come in the form of compulsions, rituals, or mental acts in response to an obsession, according to rigid rules, or to prevent feared events. In this sense, RRB in OCD involves some ‘rational’ component relating to dysfunctional beliefs (American Psychiatric Association, 2013, pp. 237–238). By contrast, RRB symptoms in ASD seem to have quite a different origin and significance within a broader picture of individual functioning. It is worth mentioning that the current diagnosis of OCD fails to fully cover the RRB symptoms as manifested in ASD (Brukner-Wertman et al., 2016).

15 Other measures of social, pragmatic, and communicative abilities are discussed in Adams (2002), Ketelaars & Embrechts (2017), Norbury (2014), Swineford et al. (2014), and Yuan & Dollaghan (2018). Here we do not review them all as the problems they face are substantially similar.

16 We will return to this point in the Conclusion, where we will discuss the possibility of considering SPCD a research entity.

17 As Weismer et al. (2021) pointed out, “the types of support and intervention services that are provided within the United States, for instance, differ substantially between these two conditions. There are various support networks and insurance waivers (Medicaid: Autism Services 2019) available for intensive […] intervention for ASD. However, services for language disorders (which is how SCD is categorized according to DSM-5) are typically intermittent and much less intense”.

18 The DSM-IV criteria of PDD-NOS state that “This category should be used when there is a severe and pervasive impairment in the development of reciprocal social interaction *or* verbal and nonverbal communication skills, or when stereotyped behavior, interests, and activities are present” (DSM-IV, p. 77, emphasis added) while those of DSM-IV-TR states that “This category should be used when there is a severe and pervasive impairment in the development of reciprocal social interaction *associated with* impairment in either verbal or nonverbal communication skills or with the presence of stereotyped behavior, interests, and activities” (DSM-IV-TR, p. 84).

19 The framing of autism-related disorders as a spectrum was also meant to accommodate the results of studies of genetic and environmental risk, which pointed at a dimensional view of psychopathology (American Psychiatric Association, 2013, p. 12; see also Jang, 2005; Knopik et al., 2017; Plomin, Haworth, & Davis, 2009; for a critical analysis, see Serpico, 2020).

20 For recent discussions on this aspect, see Chown & Leatherland (2021) and Happé & Frith (2020, 2021).

21 With regards to ASD, this point was made clear by David Kupfer in a series of interviews (Harmon, 2012; Verhoeff, 2010).

22 Notably, the question of what can constitute a threshold between normal and psychopathological states is of utmost importance to achieve a clear defintion of dimensionality. Indeed, depending on whether thresholds are understood as conventional (involving, e.g., clinical or pragmatical factors) or as ‘natural discontinuites,’ the very concept of dimension can change (for a discussion, see Serpico, 2020).

23 Note that an important piece of evidence involved in the introduction of SPCD into DSM-5 was the individuation of a class of subjects with pragmatic language impairments, but with no RRB symptoms nor phonological, structural, and semantic linguistic impairments.

24 See https://www.spectrumnews.org/opinion/viewpoint/no-one-needs-diagnosis-social-communication-disorder.

25 To take another example, other mental disorders, such as Feeding disorders, might also be considered as research entities, rather than independent nosological categories (Amoretti, 2020).

26 It should be noted that this hypothesis is by no means incompatible with the above interpretation of SPCD as a cluster of symptoms.

27 We introduced this point in Sect. 4.1.
